# Loss of WTAP Impairs Early Parthenogenetic Embryo Development

**DOI:** 10.3390/ani11061675

**Published:** 2021-06-04

**Authors:** Jindong Hao, Siyi Huang, Dongxu Wang, Yongxun Jin, Mingjun Zhang, Jiabao Zhang, Xianfeng Yu

**Affiliations:** Jilin Provincial Key Laboratory of Animal Model, College of Animal Science, Jilin University, Changchun 130062, China; haojd183@163.com (J.H.); huangsy18@mails.jlu.edu.cn (S.H.); wang_dong_xu@jlu.edu.cn (D.W.); jyx0429@126.com (Y.J.); mjzhang@jlu.edu.cn (M.Z.)

**Keywords:** m^6^A, WTAP, porcine, embryo development, parthenogenetic

## Abstract

**Simple Summary:**

Wilms’ tumor 1-associating protein (WTAP) is a key subunit of the N^6^-methyl-adenosine (m^6^A) methyltransferase complex during porcine early embryo development. However, the role of WTAP in embryonic development is still unclear. In this study, we demonstrate that WTAP plays an indispensable role in embryonic development, and the loss of WTAP will promote the apoptosis of embryonic cells, and reduce the rate and quality of embryonic development.

**Abstract:**

m^6^A is one of the most common and abundant modifications of RNA molecules present in eukaryotes. The methyltransferase complex, consisting of methyltransferase-like 3 (METTL3), METTL14, and WTAP, is responsible for the m^6^A modification of RNA. WTAP was identified as an mRNA splicing regulator. Its role as a regulatory subunit of the m^6^A methyltransferase complex in embryonic development remains largely unknown. To investigate the role of WTAP in porcine early embryonic development, si-WTAP was microinjected into porcine parthenogenetic zygotes. WTAP knockdown significantly reduced the blastocyst rate and global m^6^A levels, but did not affect the cleavage rate. Betaine was supplemented into the in vitro culture (IVC) to increase the m^6^A levels. Betaine significantly increased the global m^6^A levels but did not affect the blastocyst rate. Furthermore, the pluripotency genes, including *OCT4*, *SOX2*, and *NANOG*, were downregulated following WTAP knockdown. The apoptotic genes *BAX* and *CASPASE 3* were upregulated, while the anti-apoptotic gene *BCL2* was downregulated in WTAP knockdown blastocysts. TUNEL staining revealed that the number of apoptotic cells was significantly increased following WTAP knockdown. Our study indicated that WTAP has an indispensable role in porcine early embryonic development.

## 1. Introduction

Methylation of the adenosine base at the nitrogen-6 position (m^6^A) is one of the most common and abundant post-transcriptional epigenetic modifications of RNA in eukaryotes [1,2]. Previous studies have shown that m^6^A RNA modification is regulated by adenosine methyltransferases and demethylases [3]. The m^6^A methyltransferases (or the “writers”), including METTL3 and METTL14, methylate the N^6^ position of adenosine [4]. The m^6^A demethylases (or the “erasers”), including FTO and ALKBH5, reverse the RNA methylation process [5,6,7]. Furthermore, the m^6^A binding proteins (or the “readers”), such as YTHDF2 and YTHDC1, recognize the m^6^A sites of target mRNAs and regulate the fate of the mRNA [8,9].

WTAP was originally identified as a splicing regulator that binds to human Wilms’ tumor 1 protein [10]. It plays an important role in cell cycle progression and mammalian embryo development [11]. The involvement of WTAP in RNA methylation was first observed in studies in *Arabidopsis thaliana* and yeast [12,13]. In a recent study, WTAP was reported to be the third regulatory subunit of the m^6^A methyltransferase complex [14]. Although WTAP has no inherent methylation activity, it interacts with the METTL3–METTL14 heterodimer and synergistically forms the m^6^A methyltransferase complex to promote m^6^A methylation [15].

A growing body of evidence indicates that global mRNA m^6^A levels are associated with embryonic development [16]. Previous studies have shown that a deficiency in methyltransferases led to reduced global mRNA m^6^A levels and negatively affected embryo development in mice [17]. Knockdown of WTAP in zebrafish embryos caused defects in tissue differentiation and increased apoptosis [14]. However, the biological role of WTAP in porcine early embryo development is unknown. In the present study, we investigated the effect of WTAP on global mRNA m^6^A levels and subsequent embryonic development competence by knocking down WTAP in porcine parthenogenetic embryos. Our study demonstrates that WTAP plays an indispensable role in porcine parthenogenetic early embryo development.

## 2. Materials and Methods

All chemicals and reagents in this study were purchased from Sigma-Aldrich (St. Louis, MO, USA), unless noted otherwise.

### 2.1. Oocyte Collection and In Vitro Maturation

Porcine ovaries from slaughtered pre-pubertal gilts were obtained from a local slaughterhouse and transported to the laboratory in 0.9% saline at 35 °C within 2 h. The cumulus–oocyte complexes (COCs) were isolated from 3–8 mm antral follicles aspirated using an 18-gauge needle. COCs, with multiple layers of compact cumulus cells, were selected, and washed three times in hydroxyethyl piperazine ethane sulfonic acid (HEPES) medium with 0.1% polyvinyl alcohol (PVA, *w*/*v*) and 0.05 g/L gentamycin. The COCs were cultured in 200 mL of in vitro maturation (IVM) medium, covered with mineral oil and incubated for 42 h at 38.5 °C in an atmosphere containing 5% CO_2_ at 100% humidity.

### 2.2. Parthenogenetic Activation (PA) of Oocyte and In Vitro Culture

To obtain the porcine haploid embryos, parthenogenetic activation (PA) was used. PA was performed as described in previous reports [18]. Briefly, the metaphase-II (MII) oocytes were activated by two direct-current (DC) pulses of 120 V/mm for 60 µs in the activation medium. The activated oocytes were transferred to PZM-5 medium and cultured in an atmosphere containing 5% CO_2_ at 100% humidity. The development of the oocytes into blastocysts was examined after 6 days.

### 2.3. Microinjection of siRNA into Oocytes

Before microinjection, the oocytes were cultured to MII and subjected to parthenogenetic activation. The siRNA specific for porcine WTAP (si-WTAP) was microinjected into the cytoplasm of the zygote using a Nikon TE2000-U inverted microscope (Nikon, Tokyo, Japan) and an Eppendorf Cell Tram Vario system (Eppendorf, Hamburg, Germany). The siRNA and the negative controls were microinjected into the zygotes in the same way to serve as the negative control, while the non-injected zygotes served as the normal controls. Approximately 10 pL of siRNAs were microinjected into the zygotes at a 20 µM concentration, and the number of zygotes used was indicated in the figure. Following injection, the zygotes were transferred to PZM-5 medium until they developed into blastocysts. The siRNA specifically targeting WTAP or its non-target negative control siRNA was synthesized by Genepharma (Shanghai, China). siRNA sequence: 5′-GCAAGAGUGUACUACUCAATT-3′; negative control siRNA sequence: 5′-UUGUACUACACAAAAGVUACUG-3′.

### 2.4. Betaine Treatment

After microinjection, porcine zygotes were cultured in vitro in IVC medium supplemented with betaine (B2629, Sigma, St. Louis, MO, USA) (5 mM, 10 mM, 20 mM). The concentrations of chemical reagents were chosen first based on a previously published report [19], and then, preliminary experiments were performed to determine the optimal concentrations, which were then used in subsequent experiments.

### 2.5. Gene Expression Analysis

Total RNA was extracted from each group of blastocysts (*n* = 20) using the AllPrep DNA/RNA Micro Kit (QIAGEN, Dusseldorf, Germany) following the manufacturer’s instructions. cDNA was synthesized using the First-Strand cDNA Synthesis kit (Promega, Fitchburg, WI, USA). Quantitative real-time PCR (qPCR) was performed using the BioEasy SYBR Green I Real-Time PCR Kit (Bioer Technology, Hangzhou, China) on a BIO-RAD iQ5 Multicolor Real-Time PCR Detection System (170-9780, BIO-RAD Laboratories, Hercules, CA, USA). PCR was performed using the following parameters: initial denaturation at 95 °C for 3 min, followed by 40 cycles of denaturation at 95 °C for 10 s, annealing at 60 °C for 15 s, and extension at 72 °C for 30 s. The 2^−ΔΔCT^ method was used to determine the relative gene expression, which was then normalized to the amount of the endogenous control, GAPDH. To test the stability of GAPDH, Bio-Rad iQ5 Software was used for the analysis of amplification curves, melting curves and cycle threshold values (CT values). The experiments were performed at least in triplicate. The primer sequences used in this study are listed in Table 1.

### 2.6. Immunofluorescence Staining

Briefly, the blastocysts were washed three times in PBS–PVA. Then, the thinning of zona pellucida was performed using Tyrode’s Solution (Jisskang, Qingdao, China). The embryos were fixed with 4% paraformaldehyde for 30 min at 25 °C. Following fixation, the blastocysts were washed with PBS–PVA and permeabilized in PBS containing 0.2% Triton X-100 for 30 min. The blastocysts were then incubated in PBS containing 1% bovine serum albumin (BSA) for 1 h. Next, the blastocysts were probed with m^6^A antibodies (1:500, Abcam, Cambridge, UK) and incubated at 4 °C overnight. The blastocysts were washed with PBS three times for 10 min each followed by incubation with Alexa Fluor 488-conjugated secondary antibodies (1:1000, anti-rabbit) for 1 h at RT. The DNA was stained with 10 ng/mL Hoechst 33342 (Thermo Scientific, Waltham, MA, USA) for 15–20 min. The blastocysts were washed thrice with PBS–PVA for 10 min each, air dried, and mounted on a coverslip and a glass slide using an antifade mounting medium (BOSTER, Wuhan, China). To evaluate the average fluorescence intensity in the embryos, image analysis software (ImageJ) was used.

### 2.7. TUNEL Assay

The TUNEL assay was performed as described in previous reports [20]. Briefly, the blastocysts were fixed with 4% paraformaldehyde for 1 h at 25 °C. After fixation, the blastocysts were permeabilized by treatment with 0.1% Triton X-100 for 1 h at 37 °C. The blastocysts were washed three times in PBS–PVA and incubated in the dark for 1 h at 37 °C with TdT and fluorescein-conjugated dUTPs (In Situ Cell Death Detection kit; Roche, Mannheim, Germany). The blastocysts were then stained with 10 μg/mL Hoechst 33342 for 15 min. The blastocysts were washed thrice with PBS–PVA for 10 min each, air dried, and mounted on a coverslip and a glass slide using an antifade mounting medium (BOSTER, Wuhan, China). The number of cells in the blastocysts was analyzed by using ImageJ.

### 2.8. Statistical Analysis

The data were analyzed using Student’s t-tests with the SPSS 16.0 software (SPSS Inc., Chicago, IL, USA). A *p*-value of <0.05 was considered statistically significant. The number of embryos used for the statistics in each group of the experiment is equal to n in the figure.

## 3. Results

### 3.1. WTAP Knockdown Impairs Embryo Development

**Figure 1:**
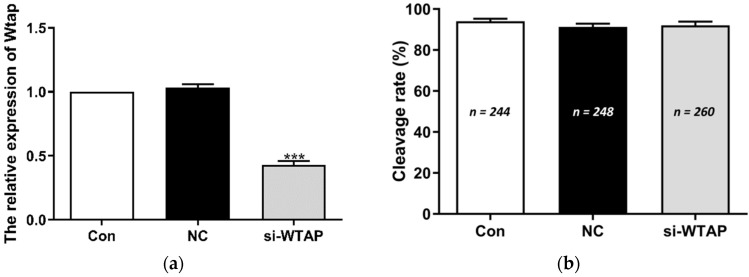

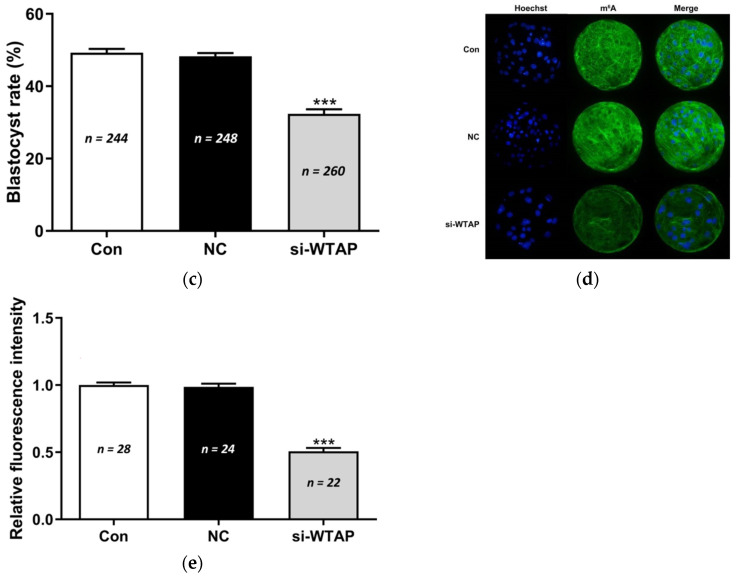
WTAP knockdown impaired embryo development and decreased m^6^A levels. (**a**) Expression of WTAP decreased significantly (*** *p* < 0.005) in the si-WTAP group compared to the Con and NC groups. (**b**) The cleavage rate in the si-WTAP group did not differ compared to other groups. (**c**) The blastocyst rates (determined on day 6) decreased significantly (*** *p* < 0.005) in the si-WTAP group compared to the other groups. (**d**) Representative images of m^6^A immunostained blastocysts in Con, NC, and si-WTAP groups. Blue, Hoechst 33342. Green, m^6^A. Merge, Hoechst 33342/m^6^A. (**e**) The relative m^6^A fluorescence intensity in blastocysts in Con, NC, and si-WTAP groups. The m^6^A expression was significantly (*** *p* < 0.005) lower in the si-WTAP group compared to the other groups. The data are presented as the mean ± S.E.M. (*n* = 4).

To investigate the role of WTAP in embryo development, si-WTAP and negative control siRNA were microinjected into zygotes. The expression of WTAP was analyzed by qPCR. The expression of WTAP was significantly (*p* < 0.005) decreased in si-WTAP-injected embryos compared to that in the negative control siRNA-injected (NC) or non-injected embryos (Con) (Figure 1a). The change in the WTAP level did not affect the cleavage rate (Con, 94.00 ± 2.89%, NC, 91.30 ± 3.47%, si-WTAP, 92.06 ± 3.98%) (Figure 1b), although it significantly (*p* < 0.005) reduced the blastocyst rate (Con, 49.28 ± 2.38%, NC, 48.28 ± 2.01%, si-WTAP, 32.38 ± 2.76%) (Figure 1c). The m^6^A expression level was analyzed using immunofluorescence (IF) staining. The results showed that m^6^A expression was significantly decreased (*p* < 0.005) in the si-WTAP group compared to the NC group and Con group (Figure 1d,e). These results indicate that WTAP knockdown reduced the global mRNA m^6^A levels and negatively affected embryo development.

### 3.2. No Effect of Betaine on WTAP-Knockdown Embryo Development

**Figure 2:**
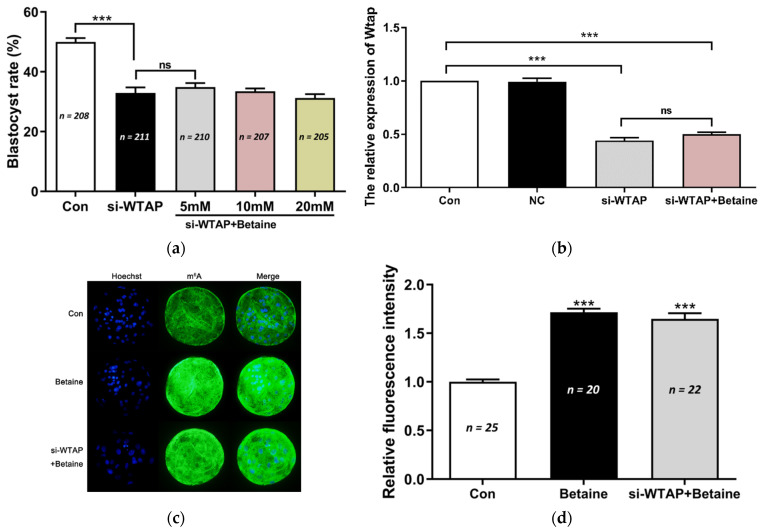
Betaine increased m^6^A levels, but had no effect on WTAP-knockdown embryo development. (**a**) Effect of betaine treatment (5 mM, 10 mM, and 20 mM) on the blastocyst rate of porcine embryos during IVC. The blastocyst rate in si-WTAP + betaine group did not show any increase compared to the Con group and si-WTAP groups. (**b**) The expression of WTAP decreased significantly (*** *p* < 0.005) in the si-WTAP + betaine group, while it did not change in the si-WTAP group. (**c**) Representative images of m^6^A immunostained blastocysts in the Con, betaine, and si-WTAP + betaine groups. Blue, Hoechst 33342. Green, m^6^A. Merge, Hoechst 33342/m^6^A. (**d**) The relative m^6^A fluorescence intensity in the blastocysts in Con, si-WTAP, and si-WTAP + betaine groups. m^6^A expression was significantly (*** *p* < 0.005) higher in the betaine and si-WTAP + betaine group compared to the Con group. The data are presented as the mean ± S.E.M. (*n* = 4). ns: no significantly.

WTAP knockdown reduced the global mRNA m^6^A levels and the blastocyst rate in porcine parthenogenetic embryos. The IVC medium was supplemented with betaine to investigate its effects on WTAP-knockdown embryo development. However, there was no change in the blastocyst rate following treatment with 5 mM, 10 mM, or 20 mM of betaine (Figure 2a). We used 20 mM of betaine for subsequent studies. The qPCR results showed that the expression of WTAP was not altered in embryos treated with betaine (Figure 2b). However, the results of the IF staining showed that m^6^A expression was significantly (*p* < 0.005) increased following treatment with betaine (Figure 2c,d). These results indicate that betaine exposure elevated the global mRNA m^6^A levels, but did not affect WTAP-knockdown embryo development.

### 3.3. WTAP Knockdown Promoted Embryonic Apoptosis

**Figure 3:**
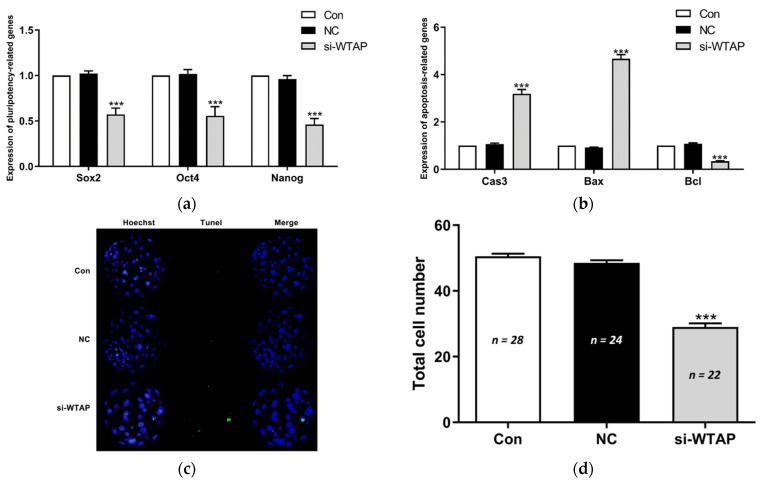
Effect of the inhibition of WTAP expression on the blastocyst quality. (**a**) The expression of pluripotency-related genes *SOX2*, *OCT4*, and *NANOG* decreased significantly (*** *p* < 0.005) in WTAP-inhibited blastocysts. (**b**) The expression of apoptotic genes *CASPASE 3* and *BAX* were significantly (*** *p* < 0.005) upregulated and the expression of the anti-apoptotic gene *BCL2* decreased significantly (*** *p* < 0.005) in the si-WTAP group. (**c**) Representative images of TUNEL-stained blastocysts in Con, NC, and si-WTAP groups. Blue, Hoechst 33342. Green, TUNEL. Merge, Hoechst 33342/TUNEL. (**d**) Average total cell count of blastocysts on day 6 in the Con, NC, and si-WTAP groups. The total number of cells decreased significantly (*** *p* < 0.005) in the si-WTAP group compared to the Con and NC groups. The data are presented as the mean ± S.E.M. (*n* = 4).

The mRNA expression of pluripotency- and apoptosis-related genes was analyzed in the blastocysts. The qPCR results showed that compared to the NC and Con groups, the expression of the pluripotent genes *SOX2*, *OCT4*, and *NANOG* were significantly (*p* < 0.005) downregulated in the si-WTAP group (Figure 3a). In addition, our study showed that the expression of the apoptotic genes *CASPASE 3* and *BAX* were significantly (*p* < 0.005) upregulated, in contrast to the expression of the anti-apoptotic gene *BCL2* (Figure 3b). To investigate the effect of WTAP knockdown on embryonic cell apoptosis, the blastocysts were analyzed by TUNEL staining. TUNEL staining showed that the number of apoptotic cells was increased following WTAP knockdown (Figure 3c). Moreover, WTAP knockdown significantly (*p* < 0.005) decreased the total number of cells in the blastocysts compared to the NC and Con groups (Figure 3d). Our results showed that the loss of WTAP promoted apoptosis in the embryos.

## 4. Discussion

The methylation of m^6^A has been shown to be a reversible process, attributable to modifications by two types of enzymes: methyltransferases and demethylases [21]. However, the identity of the enzymes responsible for each modification and the biological consequences of these modified RNAs are largely unknown [22]. A recent study showed that knockout of METTL3 reduces m^6^A in mRNAs in mice and the embryos remain in a naive state, leading to early embryonic lethality [17]. Previous studies showed that knockdown of WTAP in zebrafish embryos led to multiple developmental defects, including a smaller head and eyes, a smaller brain ventricle, and a curved notochord. Moreover, knockdown of WTAP led to a striking increase in apoptosis in zebrafish embryos [14]. In the present study, we knocked down the expression of WTAP in pig parthenogenetic embryos by microinjection of si-WTAP, which led to a reduction in the blastocyst rate. TUNEL apoptotic staining showed a significantly increased number of apoptotic cells following WTAP knockdown, which is in agreement with the study carried out on zebrafish.

**Figure 4:**
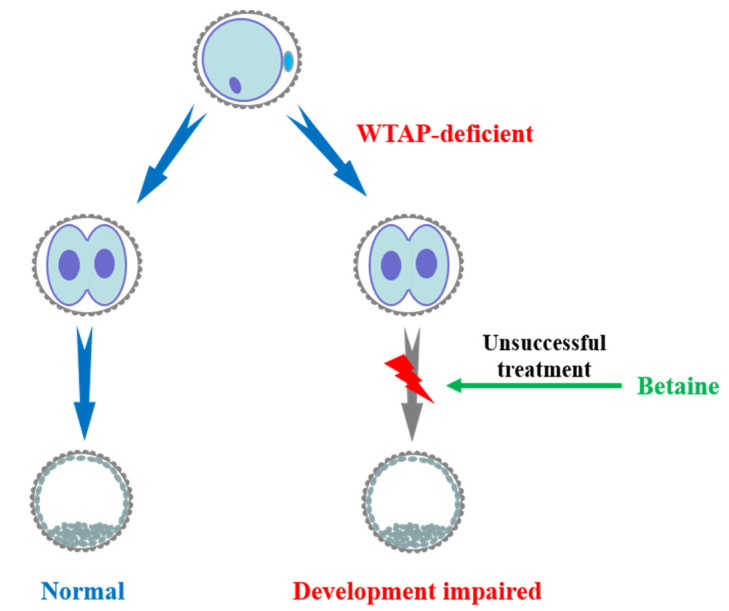
Working model showing that WTAP plays an indispensable role in porcine early embryonic development. Loss of WTAP impairs early parthenogenetic embryo development and treatment of betaine could not reverse the embryo damage.

WTAP is the third regulatory subunit in the m^6^A methyltransferase complex. Previous studies showed that METTL3 and METTL14 can interact to form heterodimers to affect m^6^A methylation [14]. WTAP interacts with the METTL3–METTL14 heterodimer and synergistically forms the m^6^A methyltransferase complex to promote m^6^A methylation. A recent study showed that reduced nucleic acid methylation could impair the maturation and development of pig oocytes [19]. Our results showed that the global mRNA m^6^A levels and blastocyst rate were reduced when we inhibited the expression of WTAP. This may suggest that RNA methylation plays an important role in both oocyte and embryonic development. Moreover, WTAP regulates transcription and alternative splicing. For example, female-lethal (2)d, a homologue of WTAP in drosophila, regulates the sex determination factor Sel by influencing the alternative splicing of pre-mRNA, and female embryos are lethal when fl(2)d is lost [23,24,25]. Previous studies have shown that betaine is usually used as a methyl donor to increase the global m^6^A level [19]. Treatment of the porcine parthenogenetic embryos with a methyl donor during IVC significantly boosted the m^6^A level within the embryos; however, the blastocyst rate and embryonic apoptosis remained unchanged. This may be because WTAP is merely a regulatory subunit without any methylation activity, and the methyl donor did not reverse the embryo damage caused by the deficiency of WTAP (Figure 4).

Previous studies showed that WTAP plays an important role in early embryo development and cell cycle regulation [11,26]. Moreover, WTAP may be associated with apoptosis. A previous study showed that WTAP activated apoptosis in smooth muscle cells by regulating the splicing of the apoptosis regulator [27]. Studies have revealed that WTAP-deficient mouse embryos failed to differentiate into the endoderm and mesoderm, and exhibited early lethality [28]. In our study, we found that the pluripotent genes *SOX2*, *OCT4*, and *NANOG* were downregulated in WTAP-inhibited blastocysts. The apoptosis genes *CASPASE 3* and *BAX* were upregulated in WTAP-inhibited blastocysts, while the anti-apoptotic gene *BCL2* showed the opposite expression pattern. We speculate that WTAP may affect the embryo development and quality of blastocysts by regulating the expression of pluripotency- and apoptosis-related genes.

## 5. Conclusions

Our study demonstrated that WTAP plays an indispensable role in regulating RNA methylation during porcine parthenogenetic embryo development. Knockdown of WTAP promoted embryonic apoptosis and negatively affected embryo development. Treatment with betaine during IVC significantly increased m^6^A levels in blastocysts, but it could not improve embryo development when WTAP was lost.

## Figures and Tables

**Table 1:** Primers used for qPCR.

Gene	GenBank Accession No.	Primer Sequences	Annealing Temperature (°C)	Product Size (bp)	Amplification Efficiency
*WTAP*	NM_001244241.1	F:GCGGGAATAAGGCCTCCAAC R:TGTGAGTGGCGTGTGAGAGA	60	136	97.4%
*OCT4*	NM001113060	F:AGTGAGAGGCAACCTGGAGA R:TCGTTGCGAATAGTCACTGC	60	166	98.2%
*SOX2*	NP_001116669.1	F:TGTCGGAGACGGAGAAGCG R:CGGGGCCGGTATTTATAATCC	60	94	97.8%
*NANOG*	NP_001123443.1	F:AGGACAGCCCTGATTCTTCCACAA R:AAAGTTCTTGCATCTGCTGGAGGC	60	198	98.4%
*CASPASE3*	NM_214131	F:GAGGCAGACTTCTTGTATGC R:CATGGACACAATACATGGAA	55	236	99.6%
*BAX*	XM_003127290	F:CGCTTTTCTACTTTGCCAGT R:GCAGAAAAGACACAGTCCAA	60	279	98.1%
*BCL2*	XM_021099593	F:CCTCCCATTTAGATGTGACTTT R:ATCCTCGATGCAGAAAAAGC	60	187	97.6%
*GAPDH*	AF017079	F:GGGCATGAACCATGAGAAGT R:AAGCAGGGATGATGTTCTGG	60	230	99.5%

## Data Availability

The data presented in this study are available on request from the corresponding author.
